# Estimating Survival in Patients with Operable Skeletal Metastases: An Application of a Bayesian Belief Network

**DOI:** 10.1371/journal.pone.0019956

**Published:** 2011-05-13

**Authors:** Jonathan Agner Forsberg, John Eberhardt, Patrick J. Boland, Rikard Wedin, John H. Healey

**Affiliations:** 1 Orthopaedic Service, Department of Surgery, Memorial Sloan-Kettering Cancer Center, New York, New York, United States of America; 2 Section of Orthopaedics and Sports Medicine, Department of Molecular Medicine and Surgery, Karolinska Institutet, Karolinska University Hospital, Stockholm, Sweden; 3 Bioinformatics Division, DecisionQ Corporation, Washington, D.C., United States of America; University of Pennsylvania, United States of America

## Abstract

**Background:**

Accurate estimations of life expectancy are important in the management of patients with metastatic cancer affecting the extremities, and help set patient, family, and physician expectations. Clinically, the decision whether to operate on patients with skeletal metastases, as well as the choice of surgical procedure, are predicated on an individual patient's estimated survival. Currently, there are no reliable methods for estimating survival in this patient population. Bayesian classification, which includes Bayesian belief network (BBN) modeling, is a statistical method that explores conditional, probabilistic relationships between variables to estimate the likelihood of an outcome using observed data. Thus, BBN models are being used with increasing frequency in a variety of diagnoses to codify complex clinical data into prognostic models. The purpose of this study was to determine the feasibility of developing Bayesian classifiers to estimate survival in patients undergoing surgery for metastases of the axial and appendicular skeleton.

**Methods:**

We searched an institution-owned patient management database for all patients who underwent surgery for skeletal metastases between 1999 and 2003. We then developed and trained a machine-learned BBN model to estimate survival in months using candidate features based on historical data. Ten-fold cross-validation and receiver operating characteristic (ROC) curve analysis were performed to evaluate the BNN model's accuracy and robustness.

**Results:**

A total of 189 consecutive patients were included. First-degree predictors of survival differed between the 3-month and 12-month models. Following cross validation, the area under the ROC curve was 0.85 (95% CI: 0.80–0.93) for 3-month probability of survival and 0.83 (95% CI: 0.77–0.90) for 12-month probability of survival.

**Conclusions:**

A robust, accurate, probabilistic naïve BBN model was successfully developed using observed clinical data to estimate individualized survival in patients with operable skeletal metastases. This method warrants further development and must be externally validated in other patient populations.

## Introduction

“Doc, how long have I got?” For the physician, such questions related to life expectancy are among the most difficult to answer. An accurate estimation of survival is important, however, and can help set patient, family, and physician expectations. Clinically, the decision to operate on patients with skeletal metastases of the extremities, as well as the choice of surgical procedure, is predicated on a patient's estimated survival [1]. The goal of surgery in this setting is not to cure the disease, but to relieve pain and optimize functional mobility for the maximum amount of time. Thus, a considerable amount of effort has been spent identifying useful prognosticators for use in the metastatic setting.

Several independent predictors of survival have been identified for patients with metastatic bone disease of the extremities [2]–[8]. These include the primary oncologic diagnosis, Eastern Cooperative Oncology Group (ECOG) performance status score [9]; number of bone metastases, presence of visceral metastases, serum hemoglobin level, senior surgeon's estimate of survival [6]; appendicular, as opposed to axial, bone metastases [10]; and type of reconstructive procedure performed [5]. Although many prognostic factors are known, the predictability of survival in patients with metastatic bone disease remains alarmingly low—only 5–15% in the best-reported series [6]. Relationships between prognostic variables are difficult to interpret, and better means of prognostication are needed since there are currently no reliable methods for estimating survival in this patient population.

Bayesian classification, which includes Bayesian belief network (BBN) modeling, is a statistical method that represents conditional, probabilistic relationships between variables, or “features.” Once described, these relationships enable the development of a graphical *n*-dimensional structure, or model, which codifies all features, including the outcome(s), into a single hierarchical network. Bayesian classification also accounts effectively for data multi-dimensionality and uncertainty—a quality that enables BBN models to maintain their robustness in the context of incomplete or discordant clinical data. As such, BBNs have been successfully used to both model complex relationships, as well as to classify outcomes, in a variety of oncologic diagnoses [11]–[20]. The purpose of this study was to determine the feasibility of developing and training Bayesian classifiers designed to estimate survival in patients undergoing surgery for skeletal metastases involving the axial and appendicular skeleton.

## Methods

After obtaining approval from the institutional review board of Memorial Sloan-Kettering Cancer Center, who issued a waiver of informed consent, we searched the institution-owned patient management database (Disease Management System, v.5.2, 1996; MSKCC) for all patients who underwent surgery for metastatic bone disease between 1999 and 2003. For each patient, we reviewed his or her medical records and imaging studies. These data, along with several other features, were used to construct the BBN model.

Fifteen candidate features were chosen based on their current clinical or historical association with survival in patients with operatively treated skeletal metastases. These included the following: age at the time of surgery, race, sex, estimated glomerular filtration rate (mL/min/1.73 m^2^), serum calcium concentration (mg/dL), serum albumin concentration (g/dL), indication for surgery (impending or completed pathologic fracture), number of bone metastases (solitary or multiple), surgeon's estimate of survival (postoperatively, in months), presence or absence of visceral metastases, presence or absence of lymph node metastases, prior chemotherapy (yes or no), preoperative hemoglobin (mg/dL, on admission, prior to transfusion, if applicable), absolute lymphocyte count (K/µL), and the primary oncologic diagnosis. The oncologic diagnosis was classified into three groups according to the method described by Katagiri et al. [21], but with some modifications. Briefly, breast, prostate, renal cell, and thyroid carcinoma, multiple myeloma, and malignant lymphoma were included in Group 3; sarcomas and other carcinomas were included in Group 2; and lung, gastric, and hepatocellular carcinoma and melanoma were included in Group 1.

The following definitions were used in this study. An impending pathologic fracture was one in which the degree of bone and/or cortical disruption warranted, in the opinion of the treating surgeon (PJB or JHH), prophylactic surgical stabilization to prevent fracture. A completed pathologic fracture was one in which a cortical lesion had caused a change in bone length, alignment, rotation, or loss of height as determined by imaging. The surgeon's estimate of survival was made preoperatively by the treating surgeon (PJB or JHH) after reviewing the patients' medical records and imaging studies, obtained a complete medical history, and performed a thorough physical examination. Biopsy-proven or clinically obvious metastases to organs within the chest or abdomen were considered visceral metastases. Only biopsy-proven metastases to the lymph nodes were considered indicative of lymph node involvement. Finally, a patient was considered as having undergone prior chemotherapy, if he or she had received any chemotherapy at any time for the current, active oncologic diagnosis.

### Conventional Statistical Analysis

Conventional statistical analysis was performed using SAS software (v.9.2, SAS Institute Inc., Cary, NC, USA). Since we modified a previously described diagnosis grouping method, overall survival of patients in each diagnosis group was compared using the Kaplan-Meier method with log-rank assessment.

### Bayesian Statistical Analysis and Model Development

The BBN models were developed using commercially available machine-learning algorithms (FasterAnalytics, DecisionQ, Washington, DC, USA) that automatically learn network structures and joint probabilities from the training data.

The training data set included all cases identified from the patient management database during the study period. All 15 candidate features were considered. Features containing continuous variables were converted into categorical variables by equal-area binning based on prior distributions. The machine-learning algorithm uses a scoring formula that balances goodness-of-fit against robustness using a parsimony metric to reduce the risk of overfitting the final model to the training data set. To refine the model, we used a step-wise training process. Quantitative and qualitative assessments were used to optimize variable preparation and selection. Unrelated and confounding features were then pruned from the preliminary models to produce the final models.

We trained the BBN to specify network structure and prior probability distributions to develop classifiers of estimated survival at 3 and 12 months, which we consider to be useful discriminators for surgical decision-making. We trained a separate classifier for each outcome measure (survival >3 months and survival >12 months) because, as a parametric modeling methodology, BBNs are not well suited to provide discrete estimates in time. Also, training two separate models eliminates the possibility of outcome measures acting as confounding features within the same BBN.

Because BBN models are directed graphs of conditional dependence, the network structure can be portrayed graphically to illustrate the conditional interdependence of the features. First-degree predictors are defined as those nodes that share edges with the outcome of interest, while second-degree predictors are those nodes that share edges with the first-degree predictors. Inference tables were calculated for both models depicting posterior estimates of probability for each possible permutation and the expected outcome, survival greater than 3 months and 12 months, respectively.

### Internal Validation

Ten-fold cross-validation was performed to assess the robustness of the final 3-month and 12-month models. Data were randomized and divided into 10 matching train-and-test sets. Each train-and-test set consisted of a training set composed of 90% of patient records and a test set composed of the remaining 10% of records. Each matching set was unique, and there was no overlap between the independent test sets. A BBN model was trained, using each training set, by applying the same parameters as the final models, then tested on the corresponding test set. A receiver operating characteristic (ROC) curve was plotted for each test to evaluate the classifier's accuracy and the model's robustness. The ROC curve is a graphical plot of sensitivity vs. 1-specificity at all discrimination threshold levels. The area under the ROC curve (AUC) was then calculated for each BBN model to assess its overall accuracy and robustness.

## Results

**Figure 1:**
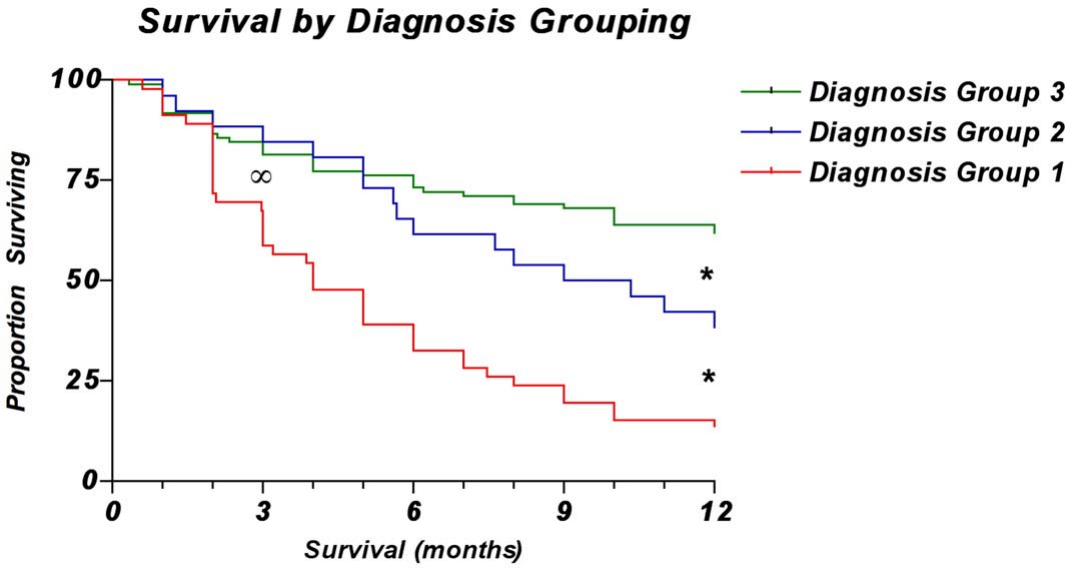
Kaplan-Meier curves showing overall survival for patients by diagnosis group. The overall survival of patients in Group 1 was significantly lower than that of patients in Groups 2 and 3 at the 3-month time point^∞^ (*p*<0.0001, log-rank test). Overall survival was significantly different between all groups at the 12-month time point* (*p*<0.0001, log-rank test).

**Figure 2:**
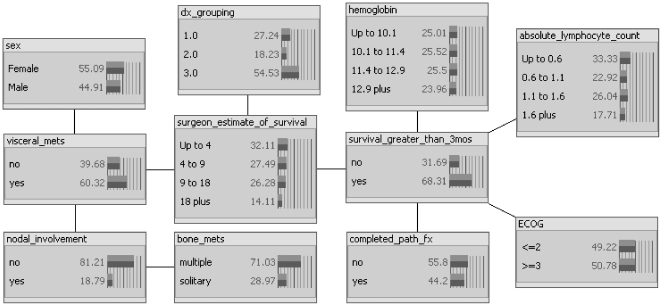
Three-month BBN model with posterior distributions depicted as proportions (%) of the training population. As shown, there are five first-degree predictors of 3-month survival: the surgeon's estimate of survival (“surgeon_estimate_of_survival”), preoperative hemoglobin concentration (“hemoglobin”), preoperative absolute lymphocyte count (“absolute_lymphocyte_count”), ECOG performance status (“ECOG”), and the presence of a completed pathologic fracture (“completed_path_fx”). The network structure indicates that the primary oncologic diagnosis (“dx_grouping”) and the presence of visceral metastases (“visceral_mets”) are both first-degree associates of the surgeon's estimate node.

A total of 189 consecutive patients were identified and, thus, used for this analysis. Median follow-up was 8 months (interquartile range [IQR] 2, 22), which was adequate for determining overall survival up to and including either the 3-month or 12-month postoperative time points. Median patient age was 62 years (54, 72). Most patients were women (55.1%), and most were white, non-Hispanic (85.2%). Most patients also had visceral metastases (60.3%), multiple bone metastases (71.0%), and prior systemic therapy (56.1%). A few patients had lymph node involvement (18.8%). When classified according to diagnosis group, most patients were in Group 3 (54.5%), followed by Group 1 (27.2%), and then Group 2 (18.2%). Regarding overall survival (Fig. 1), 58 patients (30.7%) survived less than 3 months, 53 (28.0%) survived 3–12 months, and 78 (41.3%) survived more than 12 months. Baseline (posterior) distributions represented as proportions are depicted in Fig. 2.

**Figure 3:**
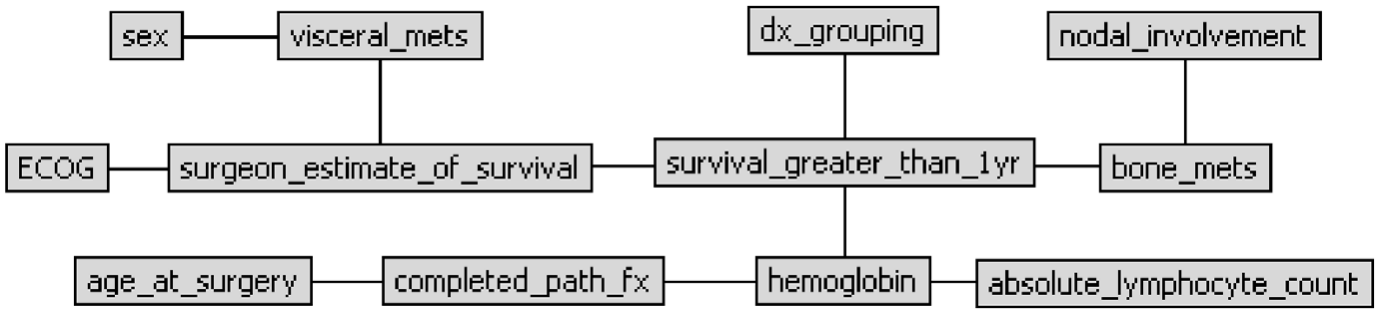
Twelve-month BBN model. As shown, there are four first-degree predictors of 12-month survival: the surgeon's estimate of survival (“surgeon_estimate_of_survival”), preoperative hemoglobin concentration (“hemoglobin”), the number of bone metastases (“bone_mets”), and the primary oncologic diagnosis (“dx_grouping”). In contrast to the 3-month model, the network structure of the 12-month model indicates that the ECOG performance status (“ECOG”) and the presence of visceral metastases (visceral_mets) are both first-degree associates of the surgeon's estimate node.

The features comprising the final models are described in Table 1. First-degree predictors differed between the two models. In the 3-month model (Fig. 2), the surgeon's estimate of survival, hemoglobin concentration, absolute lymphocyte count, completed pathologic fracture, and ECOG performance status were first-degree predictors of survival. In the 12-month model (Fig. 3), only the surgeon's estimate of survival, hemoglobin concentration, number of bone metastases, and the diagnosis group were first-degree predictors of survival. In the 3-month model, the diagnosis group and the presence of visceral metastases were first-degree associates of the surgeon's estimate node. In contrast, in the 12-month model, ECOG performance status and presence of visceral metastases were first-degree associates of the surgeon's estimate node.

**Table 1:** Network features used in the final BBN models.

Feature	Model Label	Description	Node States
Survival >12 months	survival_greater_than_1year	Overall survival exceeding 12 months	yes, no
Survival >3 months	survival_greater_than_3mos	Overall survival exceeding 3 months	yes, no
**S**urgeon's estimate of survival	surgeon_estimate_of_survival	The senior surgeon's estimate of survival(in months) after obtaining the patient's history, reviewing his or her laboratory and imaging results, and performing a thorough physical examination	<4, 4–9, 9–18, >18
Oncologic diagnosis grouping	dx_grouping	Primary oncologic diagnosis, grouped as follows:1: lung, hepatocellular, and gastric carcinoma; melanoma2: sarcoma and other carcinoma, not in Groups 1 or 33: breast, prostate, thyroid, and renal cell carcinoma; myeloma; lymphoma	1, 2, 3
ECOG performance status	ECOG	Eastern Cooperative Oncology Group performance status, assessed preoperatively by treating physician	≤2, ≥3
Pathologic fracture status	completed_path_fx	Indicates whether surgery was performed for an impending or completed pathologic fracture	yes, no
Skeletal metastases	bone_mets	Indicates whether the patient had solitary or multiple skeletal metastases	solitary, multiple
Organ metastases	visceral_mets	Presence of metastases to visceral organs, lungs, or brain	yes, no
Lymph node metastases	nodal_involvement	Presence of lymph node metastases	yes, no
**Sex**	sex	Patient sex	male, female
Hemoglobin **concentration**	hemoglobin	Preoperative hemoglobin concentration (in mg/dL), prior to blood transfusion, if applicable	<10.1, 10.1–11.411.4–12.9, >12.9
Absolute lymphocyte count	absolute_lymphocyte_count	Preoperative absolute lymphocyte count (in mg/dL), prior to transfusion, if applicable	<0.6, 0.6–1.1, 1.1–1.6, >1.6

Features included in the final BBN models. Each feature, its label, description, and possible node states are shown. Continuous variables are represented as categorical variables.

Cross-validation ROC curve analysis showed that both the 3-month and 12-month models were robust. Mean AUC for 3-month probability of survival was 0.85 (95% CI: 0.80–0.93), and mean AUC for 12-month probability of survival was 0.83 (95% CI: 0.77–0.90). Inference tables were calculated for 3-month survival (Table 2) and 12-month survival (Table 3). Posterior estimates of probability of 3-month survival ranged from 0.8–96.7%, while posterior estimates of probability of 12-month survival ranged from 0.9–99.2%. In each case, we used the estimated case frequency to select the ten most likely inferential cases, since there were 256 and 128 potential permutations for the 3- and 12-month models respectively.

**Table 2:** Posterior estimates of survival at 3 months (10 most frequent cases).

	Drivers					Target	
Expected frequency	ECOG	Absolute lymphocyte count (K/µL)	Completed Pathologic Fracture	Hemoglobin concentration (mg/dL)	Surgeon's estimate of survival (months)	Probability of survival >3 months	
						No	Yes
2.02%	≥3	<0.6	Yes	<10.1	<4	96.7	3.3
1.33%	≥3	<0.6	No	<10.1	<4	91.1	8.9
1.73%	≥3	<0.6	Yes	10.1–11.4	<4	95.3	4.7
1.17%	≥3	<0.6	No	10.1–11.4	<4	87.6	12.4
1.09%	≥3	0.6–1.1	Yes	<10.1	<4	94.8	5.2
0.95%	≥3	0.6–1.1	Yes	10.1–11.4	<4	92.7	7.3
0.90%	≤2	<0.6	Yes	<10.1	<4	89.5	10.5
0.87%	≥3	<0.6	Yes	11.4–12.9	<4	86.5	13.5
0.81%	≤2	1.1–1.6	No	>12.9	4–9	0.8	99.2
0.80%	≤2	<0.6	Yes	10.1–11.4	<4	85.6	14.4

The 3-month posterior estimates of survival characterizing the data set by most- to least-frequent cases. The ten most likely cases were selected from 256 possible permutations.

**Table 3:** Posterior estimates of survival at 12 months (10 most frequent cases).

		Drivers			Target	
Expected frequency	Number of bone metastases	Diagnosis Group	Hemoglobin concentration (mg/dL)	Surgeon's estimate of survival (months)	Probability of survival >12 months	
					No	Yes
3.12%	Multiple	3	<10.1	<4	94.4	5.6
3.08%	Multiple	3	10.1–11.4	<4	93.3	6.7
2.94%	Multiple	1	<10.1	<4	99.2	0.8
2.92%	Multiple	3	>12.9	9–18	16.2	83.8
2.87%	Multiple	1	10.1–11.4	<4	99.1	0.9
2.46%	Multiple	3	10.1–11.4	4–9	75	25
2.41%	Mutiple	3	≤10.1	4–9	78.4	21.6
2.21%	Multiple	3	11.4–12.9	<4	80.7	19.3
2.09%	Mutiple	3	10.1–11.4	9–18	49.3	50.7
2.02%	Solitary	3	11.4–12.9	9–18	6.4	93.6

The 12-month posterior estimates of survival characterizing the data set by most- to least-frequent cases. The ten most likely cases were selected from 128 possible permutations.

## Discussion

Because predictive models permitting individualized estimation of survival are lacking, we trained two full machine-learned BBN models, using observed clinical data, to estimate survival in patients with operable skeletal metastases. These models were shown to be robust on ten-fold cross-validation. We also characterized the importance of the surgeon's estimate of survival, and we believe that it should be included in future iterations of this model, whenever possible. However, if the surgeon's estimate is not included, ECOG performance status, the oncologic diagnosis group, and the presence of visceral metastases may be used to estimate this important subjective feature.

In the present analysis, we used the 3-month and 12-month time points, since we consider them to be useful discriminators for surgical decision-making. We believe that survival less than 3 months is a relative contraindication to surgical management of certain impending pathologic fractures, particularly those in the upper extremity. If surgical stabilization is deemed to be necessary, shorter life expectancies (3–12 months) are thought to warrant less-invasive stabilization procedures that do not require prolonged rehabilitation periods. On the other hand, longer life expectancies (≥12 months) warrant more-durable reconstruction procedures, which are associated with significant operative morbidity and longer rehabilitation times. Thus, less-invasive stabilization techniques may be appropriate for patients with an estimated survival of less than 12 months, while more-durable reconstruction options may be considered for those with an estimated survival longer than 12 months. These concepts and the surgical techniques are described elsewhere [22].

Unfortunately, although modeling and, thus, estimating survival in patients with metastatic disease is important in the surgical management of these patients, it has proven to be challenging [2], [5], [6], [8]. For instance, there is no consensus regarding which variables should be included in survival estimation models. In an attempt to codify several known independent predictors of survival, Nathan et al. developed a conventional, regression-derived nomogram based on 191 patients with operable skeletal metastases [6]. Variables, collected prospectively, included ECOG performance status, number of bone metastases, presence of visceral metastases, and serum hemoglobin concentration. Evaluation in a five-patient test set yielded an accurate survival estimate in two patients, but this method has not yet been internally or externally validated.

A closer look at Nathan et al.'s results gives us some insight into the challenges inherent to predicting survival in these patients [6]. Linear regression analysis revealed that, in terms of accuracy, the “attending surgeon's [subjective] prediction” of survival was superior to any other covariate included in their analysis. The authors attributed this finding to the attending surgeon's extensive experience in the field, but no further conclusions were drawn. Other studies, including a systematic review of physicians' survival predictions, also emphasize subjective assessments and cite the importance of the physician's role in estimating patient survival [23]–[25].

In the context of the current study, what makes the surgeon's estimate of survival so important? Clearly, an estimate of survival is made after reviewing the patient's medical records and imaging studies and performing a thorough physical examination on the patient. Some variables considered by the surgeon are objective and thus quantifiable, such as ECOG performance scores, laboratory results, and radiographic findings. However, others are subjective and thus not quantifiable, such as the patient's appearance of sickness, the patient's demeanor, and the surgeon's “gut feeling” following the consultation. These subjective observations are unlikely to be useful as individual covariates in the traditional sense, but we believe that they are contained within the surgeon's estimate, which was found to be an important first-degree predictor in both survival models (Figs. 2 and 3). Historically, the physician's assessment appears to be important when estimating survival [6], [23]–[25], and if the subjective observations listed above are indeed represented by the surgeon's estimate, the results of the present study support this claim.

Fortunately, the BBN method may help us to better understand the complex relationships that exist between objective and subjective features. The hierarchy of conditional dependence, identified by the BBN, defines how individual features known to be associated with survival relate to one another. When graphically displayed, the network structure depicts these relationships. For example, we found that the surgeon's estimate is a first-degree predictor of overall survival at both 3 months and 12 months. However, the nodes associated with the surgeon's estimate differed between the models. In the 3-month model, the oncologic diagnosis group and the presence of visceral metastases were first-degree associates of the surgeon's estimate node. In contrast, in the 12-month model, ECOG performance status and the presence of visceral metastases were first-degree associates. This suggests that in short-term survivors, represented by the 3-month model, specific oncologic diagnosis and the presence of visceral metastases are most influential in the development of a survival estimate by the senior surgeon, while in longer-term survivors, represented by the 12-month model, ECOG performance status and the presence of visceral metastases are most important in the development of the surgeon's estimate of survival. Other features, such as the number of bone metastases, hemoglobin concentration, absolute lymphocyte count, and completed pathologic fracture, may also inform the surgeon's estimate, but they have significant prognostic value of their own, as shown in both model structures (Figs. 2 and 3). This is further supported by the observation that a BBN model that included the surgeon's estimate and these independent factors (the number of bone metastases, hemoglobin concentration, absolute lymphocyte count, and completed pathologic fracture) outperformed a BBN model containing the surgeon's estimate alone (data not shown). Nevertheless, in order to capture the subjective, yet important, elements of the clinical evaluation, we believe that future predictive models designed to estimate survival in patients with operatively treated skeletal metastases should include a surgeon's estimate of survival.

This cohort of surgically treated patients with metastatic disease involving the axial and appendicular skeleton is among the largest reported in the literature [1], [5], [6], [10], [21]. As such, it is well suited for prognostic model development. Nevertheless, the limitations of this study are those inherent to a feasibility study. No comparisons were made to existing models or nomograms, and no external validation was performed. Validation of these models in an independent data set followed by an appropriate comparison to existing models is the next logical step in the evaluation of this method. The ultimate goal being to provide an accurate, personalized answer to the difficult question, “Doc, how long have I got?”
